# A standardised approach to systematically select risk-outcome pairs in environmental burden of disease research

**DOI:** 10.1186/s12963-026-00514-0

**Published:** 2026-09-21

**Authors:** Tessa M.I. Haverkate, Brecht Devleesschauwer, Gunn Marit Aasvang, Ricardo Assunção, Anette K. Bølling, Susanne Breitner-Busch, Jurgen Buekers, Alberto Castro, Vanessa Gorasso, Hamid Y. Hassen, Carla Martins, Periklis Charalampous, Juanita A. Haagsma

**Affiliations:** 1 Department of Public Health, Erasmus MC, University Medical Center Rotterdam, Rotterdam, The Netherlands; 2 Department of Epidemiology and Public Health, Sciensano, Brussels, Belgium; 3 Department of Translational Physiology, Infectiology and Public Health, Ghent University, Merelbeke, Belgium; 4 Department of Air Quality and Noise, Norwegian Institute of Public Health, Oslo, Norway; 5 Food and Nutrition Department, National Institute of Health Dr. Ricardo Jorge, Lisbon, Portugal; 6 Egas Moniz Center for Interdisciplinary Research (CiiEM), Egas Moniz School of Health & Science, Almada, Portugal; 7 Institute for Medical Information Processing, Biometry, and Epidemiology, Faculty of Medicine, LMU Munich, Munich, Germany; 8 Institute of Epidemiology, Helmholtz Zentrum München - German Research Center for Environmental Health, Neuherberg, Germany; 9 Environmental Intelligence Unit, Flemish Institute for Technological Research (VITO), Mol, Belgium; 10 Swiss Tropical and Public Health Institute, Allschwil, Switzerland; 11 University of Basel, Basel, Switzerland; 12 Public Health Research Centre, Comprehensive Health Research Center, NOVA National School of Public Health, CHRC, REAL, NOVA University Lisbon, Lisbon, CCAL Portugal; 13 Institute of Health and Society (IRSS), Université catholique de Louvain, Brussels, Belgium

**Keywords:** Environmental burden of disease, Risk–outcome pairs, Disability-adjusted life years, Environmental health

## Abstract

**Background:**

Environmental Burden of Disease (EBoD) quantifies health impacts attributable to environmental risk factors. The methodological choice on which risk-outcome pairs to include in EBoD studies is technically and conceptually demanding, with diffuse exposures and complex causal chains. This makes transparent criteria for inclusion of risk–outcome pairs essential. In response, we developed a standardised approach for systematically and transparently selecting risk-outcome pairs within environmental burden of disease research.

**Methods and results:**

The standardised approach was developed through expert elicitation and iterative discussions, ultimately resulting in five sequential steps designed to strengthen consistency and transparency: (1) define and characterise the exposure to the environmental risk factor; (2) identify potential health outcomes from the literature; (3) evaluate the strength of evidence criteria according to set criteria; (4) select risk-outcome pairs based on strength of evidence; and (5) select exposure-response functions.

**Conclusions:**

By making underlying assumptions explicit, the proposed approach to the selection of risk-outcome pairs can reduce subjectivity in the selection process and support more consistent, evidence-based decisions.

## Introduction

The burden of disease (BoD) provides a framework for measuring and comparing population health, expressed through the Disability-Adjusted Life Year (DALY) metric, which captures both mortality and morbidity [1]. Environmental Burden of Disease (EBoD) quantifies health impacts attributable to environmental risk factors (e.g. transportation noise). The field of EBoD has gained importance over the past decades due to a rising awareness of environmental health risks and the need to assess the health impact of policies [2]. A major methodological choice is to decide which risk-outcome pairs to include in BoD studies [3]. This decision directly determines which health impacts are counted and how comprehensive the resulting burden estimate is.

Selecting risk-outcome pairs in EBoD is technically and conceptually demanding. Environmental risk factors can involve diffuse exposures that vary across time and space and act through complex causal chains. In addition, the relevance of a risk-outcome pair may depend on the exposure source, timeframe, population, and geographical setting, underscoring the need to clearly define these aspects of exposure. Similarly, conceptual choices about which types of health effects are considered relevant shape which outcomes are eligible for selection. The considered health effects influence how the resulting risk-attributable burden estimates should be interpreted, particularly when relevant effects are not captured by conventional disease classifications.

Evidence for many environmental risks is still emerging [4, 5], meaning that reliance on previous assessments alone may produce outdated or incomplete evaluations, potentially overemphasising some health outcomes while underrepresenting others. Available studies can differ substantially in their exposure definitions, outcome definitions, study designs, and measures of disease frequency, making transparent criteria for identifying and selecting risk-outcome pairs essential. Differences in the selection of exposure-response functions, or in the methods used to derive them, can also influence the resulting risk-attributable burden estimate [6, 7].

Given that environmental exposures affect entire populations, decisions about which risk–outcome pairs to include carry significant implications for the estimated disease burden and the policy priorities derived from it. This paper outlines a standardised approach for systematically and transparently identifying, evaluating, and selecting risk-outcome pairs and corresponding exposure-response functions within EBoD research to guide public health researchers, data analysts, and students.

## Methods and results

### Methodological aspects

The formulation of the steps was informed by expert elicitation and iterative discussions. These steps were developed within the BEST-COST project [8], which aims to develop a consensus methodological framework for estimating EBoD. Between 27 January and 13 April 2023, a series of a total of five online meetings was held with, on average, ten participating experts from the BEST-COST project team. Participants had expertise in burden of disease research and methods, environmental epidemiology, exposure assessment, and environmental health. During these meetings, participants discussed methodological considerations relevant to the selection of risk-outcome pairs. Meeting minutes were taken to document all discussions and decisions. The resulting steps were further refined through iterative feedback from the participating experts and co-authors.

The following steps are illustrated using *environmental noise pollution* as a practical example, which was developed as part of the project’s work. This practical example is used to show how the approach can guide the identification, evaluation, and selection of risk-outcome pairs.

### Guiding steps to systematically select risk-outcome pairs in EBoD

#### Step 1: Define and characterise the exposure to the risk factor

The nature of exposure to the environmental risk factor should be clearly defined, according to the scope and aim of the intended EBoD assessment. This definition should specify the characteristics of exposure relevant to the assessment, without restricting the evidence review to studies that use the same exposure assessment method. Key aspects include: (i) the exposure source, such as transportation noise; (ii) the exposure timeframe, such as short-term (i.e. daily) or long-term (i.e. yearly) exposure); (iii) the geographical or contextual scope of the assessment, for instance a national, regional, or global assessment, including ecological and climatic context where relevant; and (iv) the population and exposure setting, such as residential exposure in the general adult population or occupational exposure.

In the practical example used throughout these steps, we selected the following exposure scenario: long-term residential exposure to environmental transportation noise, including road traffic noise, railway noise, aircraft noise, in the general population at the global level.

#### Step 2: Identify potential health outcomes from the literature

Potential health outcomes associated with the exposure should be identified from the literature. The aim of this step is to create an initial inventory of health outcomes that might follow from exposure to the risk-factor and could therefore form potential risk-outcome pairs. Potential health outcomes can be drawn from synthesised sources, such as systematic reviews, meta-analyses, meta-regressions, or authoritative reports from institutions such as the World Health Organisation (WHO), topic centres of the European Environment Agency (EEA), and national public health agencies [4, 5]. In the absence of recent publications on the risk factor, conducting a scoping review or systematic literature review is recommended.

Researchers should define, justify, and document the conceptual boundaries used to determine when an outcome is considered potentially relevant for the assessment. This may include whether the assessment will consider direct effects, indirect effects, or mediated pathways and whether outcomes need to be defined as clinical diagnoses or may also include symptoms or functional outcomes. While International Classification of Diseases (ICD) codes could help define and demarcate the outcome more precisely, the absence of an ICD code does not inherently preclude a condition from being considered as a health outcome [1, 9]. At this stage, all available metrics of disease frequency should be considered to avoid premature exclusion when outcome measures vary across the literature.

In the example of long-term residential exposure to transportation noise in the general population, potential health outcomes may be identified form sources such as the WHO Environmental Noise Guidelines, systematic reviews, and pooled cohort studies on transportation noise [10–14]. In this example, the conceptual boundaries are defined to include both clinical outcomes and non-clinical health outcomes. This allows noise annoyance and sleep disturbance to be considered, even when they are not defined by and mapped to an ICD code. Other potential health outcomes that may be identified based on these sources, include ischemic heart disease, stroke, hypertension, diabetes, cognitive impairment, and mental health outcomes.

#### Step 3: Evaluate the strength of evidence according to pre-defined criteria

For each potential risk-outcome pair identified in Step 2, the available evidence should be assessed to determine whether it supports an association between the risk factor and the health outcome. Clear criteria for the strength of evidence must be defined before selecting health outcomes, to ensure decisions are transparent and reproducible. In environmental health, cohort or case-control studies, supported by exposure–response data, can provide sufficient evidence without the need for experimental trials. Evaluating causality may involve integrating findings from epidemiological studies, in vivo and in vitro research, in silico models, and adverse-outcome-pathway analysis. Examples of relevant criteria include consistency of findings, study quality, biological plausibility and specificity of observed associations. Evidence-grading frameworks (e.g. World Cancer Research Fund criteria or the Integrated Science Assessments) offer transparent methods for evaluating the body of evidence and determining the level of confidence in the association between a given risk factor and health outcome [15, 16]. Depending on the framework used, the outcome of this step may be an evidence classification, such as sufficient, limited, or insufficient evidence, or another structured judgement on the certainty or strength of the evidence. This classification should then inform the selection of risk-outcome pairs in Step 4. Context-specific methodological choices are also required, such as determining how to weigh emergent findings, interpret heterogeneous study designs and exposure metrics, and account for the typically small effect sizes encountered in environmental health.

For the practical example, transportation noise in the general population, the strength of evidence for each potential risk-outcome pair was evaluated using the strength of the association, informed by the GRADE (Grading of Recommendations Assessment, Development and Evaluation) quality-of-evidence methods and criteria, and biological plausibility [17]. For each potential risk-outcome pair identified in Step 2, we summarised the available evidence and assigned evidence judgement according to these criteria.

#### Step 4: Select risk-outcome pairs based on strength of evidence

The aim of this step is to determine which risk-outcome pairs will be included in the EBoD assessment, using the evidence classification obtained in Step 3 as the basis for decision-making. Researchers should explicitly define the inclusion criteria, for example by selecting only risk-outcome pairs for which the evidence is classified as “convincing” and “probable”, depending on the framework used in Step 3. In addition, potential overlap between selected outcomes should be assessed to avoid double counting, particularly when both intermediary and downstream outcomes are considered.

**Fig. 1:**
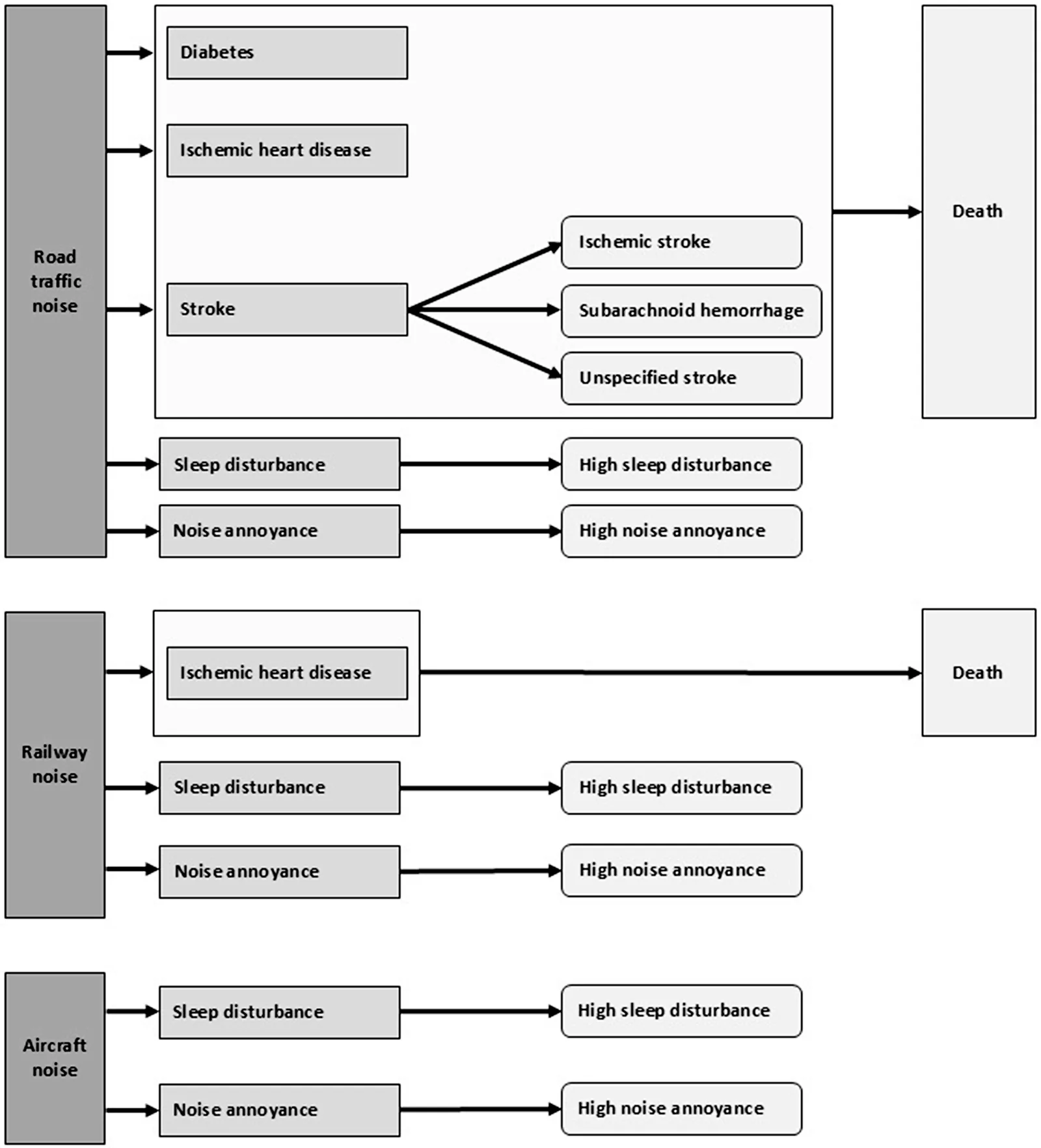
Schematic representation of selected transportation noise risk-outcome pairs. *Note*. A schematic representation (also referred to as a disease model or outcome tree) of the risk-outcome pairs selected in the practical example of long-term residential exposure to transportation noise. Dark grey boxes indicate transportation noise sources, medium grey rectangular boxes indicate selected health outcomes, and light grey rounded boxes indicate health states

In the example of transportation noise, risk-outcome pairs were selected when evidence met the predefined causality criteria (see Step 3), for at least one transportation noise source. Based on this, selected outcomes included cause-specific mortality, incidence of diabetes, ischemic heart disease and stroke, cognitive impairment, prevalence of sleep disturbance, and prevalence of noise annoyance (Fig. 1).

#### Step 5: Select exposure-response functions

For each selected risk-outcome pair, an appropriate exposure-response function should be identified to quantify the population attributable fraction (PAF) [3]. Exposure-response functions can either be obtained from existing literature or generated using established synthesis [18] or the conventional meta-regression. The selected function should be compatible with the risk-outcome pairs selected in Step 4. When multiple exposure-response functions are available (e.g. regional- or global-level), researchers should justify their choices based on criteria such as the quality [6, 19]. If an exposure-response function no longer reflects available evidence, researchers can either derive an updated function by incorporating recent epidemiological studies through a systematic review and quantitative synthesis or apply the existing function while explicitly addressing any limitations in the resulting risk-attributable disease burden estimates.

In the environmental transportation noise example, exposure-response functions should be selected separately for each selected health outcome and for each transportation noise source [7]. For example, an exposure-response function for ischemic heart disease derived for road traffic noise should not automatically be applied to railway noise.

After completion of these steps, the selected risk-outcome pairs and corresponding exposure-response functions can be used in the subsequent burden calculation, including estimation of PAFs and calculations of DALYs, as described in existing stepwise guidance for DALY calculations [20].

## Discussion

This paper provides a structured approach to support transparent decision-making when identifying, evaluating, and selecting risk-outcome pairs and corresponding exposure-response functions within EBoD research.

Existing EBoD assessments differ in their approaches to selecting risk-outcome pairs. Examples are the approach that is taken in the Global Burden of Disease (GBD) study versus the approach that is used by WHO. For risk-outcome pairs already included in the GBD assessments, eligibility was based on convincing or probable evidence of an association, assessed using the World Cancer Research Fund methods and criteria [21]. New risk-outcome pairs considered for inclusion in the GBD are further evaluated based on informed expert judgement, including their potential contribution to risk-attributable disease burden, as well as their relevance to public health policy. In addition, to ensure comparability of the resulting (environmental) risk-attributable estimates, the health outcomes included in GBD assessments must be defined and mapped to the ICD cause list. WHO instead assesses each risk factor independently, applying the GRADE quality-of-evidence methods and criteria to specific noise-outcome and pollutant-outcome pairs [10, 17, 22]. Because the WHO approach is not restricted to the ICD-mapped cause list, it can evaluate broader environmental health outcomes (e.g. aircraft noise-sleep disturbance). In addition, national-level EBoD assessments often do not undertake an independent, systematic assessment of evidence when selecting risk-outcome pairs. For instance, the Nordic assessment of transportation noise selected noise annoyance, sleep disturbance, and ischemic heart disease as health outcomes largely on the basis of the existing WHO evidence synthesis and available exposure-response functions [9]. A similar approach has been used in Canadian air-pollution disease burden estimates, where a predefined set of health outcomes and exposure-response functions was adopted on the basis of expert-panel consensus [23]. It should be noted that the aim of our proposed approach (Steps 1–5) is not to replace these existing approaches; rather, it can be used to help researchers document and justify how and why they made certain choices regarding risk-outcome pairs in their EBoD studies. By providing guidance on these methodological decisions, the proposed approach complements reporting guidance such as STROBOD [24]. By supporting a more reproducible workflow, the approach can improve transparency in EBoD assessments and help users interpret differences between risk-attributable burden estimates.

Although the proposed approach provides a structured process for selecting risk-outcome pairs, several methodological challenges remain when applying it in practice. First, double counting may occur when selected health outcomes lie on the same causal pathway or when exposures are correlated and co-occur [3]. Although Step 4 explicitly includes the need to assess potential overlap between selected outcomes, this issue is not inherently resolved by the proposed approach. Rather, the approach helps to make such decisions explicit and encourages researchers to document how potential overlap was assessed and addressed. A schematic representation of the selected health outcomes and their health states, also referred to as a disease model, is recommended to depict each risk-outcome pair, including the distinct health states of each health outcome, and the possible transitions between them, culminating in recovery, long-term disability, or death [24]. While this may clarify how health outcomes relate to one another, and where potential overlap may occur, such schematic representations do not replace formal modelling decisions. Second, after risk-outcome pairs are selected, their burden estimates depend on the selected exposure-response function. The applicability of a given function may depend on the exposure levels in the target setting, adjustment for relevant confounders, and the modelling method used to derive the function [7]. Finally, while strict evidence thresholds may improve the validity and credibility of risk-attributable disease burden estimates, they may also contribute to the under-representation of emerging or under-researched risk-outcome pairs. Exclusion of risk-outcome pairs with insufficient evidence from risk-attributable disease burden assessments can obscure their potential public health relevance and, in turn, may limit incentives for further research. This creates a risk of reinforcing existing evidence gaps. Uncertain risk-outcome pairs should be transparently documented and, where appropriate, considered for sensitivity analyses or qualitative discussion.

## Conclusions

By making underlying assumptions explicit, this proposed approach can reduce subjectivity in the process of selecting of risk-outcome pairs makes and support more consistent, evidence-based, and transparent decisions. EBoD studies that reflect up-to-date evidence on existing and emerging environmental challenges are crucial for monitoring the impact of environmental risks on public health and guiding environmental policy amid global environmental changes.

## Data Availability

No datasets were generated or analysed during the current study.

## Abbreviations

DALY: Disability-Adjusted Life Year
EBoD: Environmental Burden of Disease
WHO: World Health Organisation
ICD: International Classification of Diseases
GRADE: Grading of Recommendations Assessment, Development and Evaluation
EEA: European Environment Agency
PAF: Population Attributable Fraction
GBD: Global Burden of Disease
STROBOD: Standardised Reporting of Burden of Disease studies
